# Case Report: A neonatal case of cryopyrin-associated periodic syndrome with severe funisitis and neonatal asphyxia

**DOI:** 10.3389/fped.2024.1397412

**Published:** 2024-05-14

**Authors:** Yuri Hayashida, Maho Hatano, Kazuyuki Ito, Manabu Sugie, Junko Kunieda, Masaki Shimizu, Tomohiro Morio, Chikako Morioka

**Affiliations:** ^1^ Department of Pediatrics, Tokyo Medical and Dental University Hospital, Tokyo, Japan; ^2^ Department of Comprehensive Pathology, Tokyo Medical and Dental University Hospital, Tokyo, Japan

**Keywords:** cryopyrin-associated periodic fever syndrome, neonatal asphyxia, umbilical cord rupture, funisitis, CINCA syndrome/NOMID

## Abstract

Cryopyrin-associated periodic syndrome (CAPS) is a genetic disorder and autoinflammatory disease characterized by chronic inflammation throughout the body. The most severe form of CAPS, Chronic Infantile Neurologic Cutaneous, and Articular (CINCA) syndrome, also known as Neonatal Onset Multisystem Inflammatory Disease (NOMID), has three main features: skin rash, CNS involvement, and joint symptoms. Although these symptoms are typically reported shortly after birth, there have been a few reports of prenatal inflammation. Here, we report our experience managing a case of a CAPS infant born in severe neonatal asphyxia due to a ruptured cord associated with severe funisitis. The baby was born at 38 weeks and 6 days of gestation, weighing 2,898 g, through an ultra-emergency Caesarian section prompted by variable deceleration. The Apgar score was 1 point at 1 min and 4 points at 5 min, necessitating intensive care due to hypoxic-ischemic encephalopathy. Upon delivery, it was observed that the umbilical cord had partially ruptured at the site of attachment to the baby, accompanied by arterial hemorrhage. Umbilical cord rupture was considered to be the cause of the sudden decrease in fetal heart rate. Pathological examination also showed that the inflammation of the cord was more severe on the side attached to the fetus and on the arterial side, suggesting that the inflammation had extended from the fetus. The father carried a genetic mutation associated with CINCA syndrome/NOMID (*NLRP3* c.2068G>A p.Glu690Lys Hetero), which was also found in the child. Histopathologic examination of the placenta and umbilical cord can provide crucial insights into the intrauterine onset of inflammation, which is the first manifestation of CINCA syndrome/NOMID in newborns. It should be noted that births with a genetic predisposition to CAPS may have complications related to the placenta and umbilical cord.

## Introduction

Cryopyrin-associated periodic syndrome (CAPS) is an autoinflammatory disorder that causes chronic inflammation throughout the body, resulting from the overproduction of IL-1β. This overproduction is driven by inflammasome-mediated activation of procaspase-1 due to abnormal cryopyrin function. The nucleotide-binding domain, leucine-rich repeat family, pyrin domain containing 3 (*NLRP3*) gene, which codes for cryopyrin, has been identified as the causative gene, inherited in an autosomal manner (1, 2). CAPS has been reported to cause mild Familial Cold Autoinflammatory syndrome (FCAS), moderate Mucke-Wells syndrome (MWS), and severe Chronic Infantile Neurologic Cutaneous, and Articular (CINCA) syndrome, also known as Neonatal Onset Multisystem Inflammatory Disease (NOMID). However, distinguishing between the disease sub-types can be challenging in some cases (3). In severe cases of CINCA syndrome/NOMID, the three main features include a skin rash, central nervous system involvement, and joint symptoms, typically presenting shortly after birth. Preterm delivery, fetal growth retardation, and excess amniotic fluid are associated with CINCA/NOMID syndrome but reports of placental or umbilical cord abnormalities in association with this condition are rare (4). In this report, we describe a case of CAPS presumably caused by inflammation and rupture of the umbilical cord, caused by inflammatory spillover from the fetus.

## Case presentation

The mother, aged 28, had experienced two pregnancies and one prior delivery. Her medical history was unremarkable, with no abnormalities or complications reported. The father had been diagnosed with Muckle-Wells syndrome in his childhood and had been receiving treatment with canakinumab. The mother conceived naturally, and no abnormalities were detected during prenatal checkups. At 38 weeks of gestation, she began experiencing labor pains and was hospitalized for management. During the first stage of labor, a sudden decrease in the fetal heartbeat was observed, and the baby was born via ultra-emergency cesarean section.

The baby was born at 38 weeks and 6 days, with a birth weight of 2,898 g (−0.49 SD), a height of 49.4 cm (+0.36 SD), and a head circumference of 34.8 cm (+1.23 SD), indicating healthy fetal growth. The Apgar scores were 1 point at 1 min, 4 points at 5 min, and 7 points at 10 min, suggesting severe neonatal distress. The cord blood pH was 6.904, with a BE of −17.6 mmol/L.

**Figure 1:**
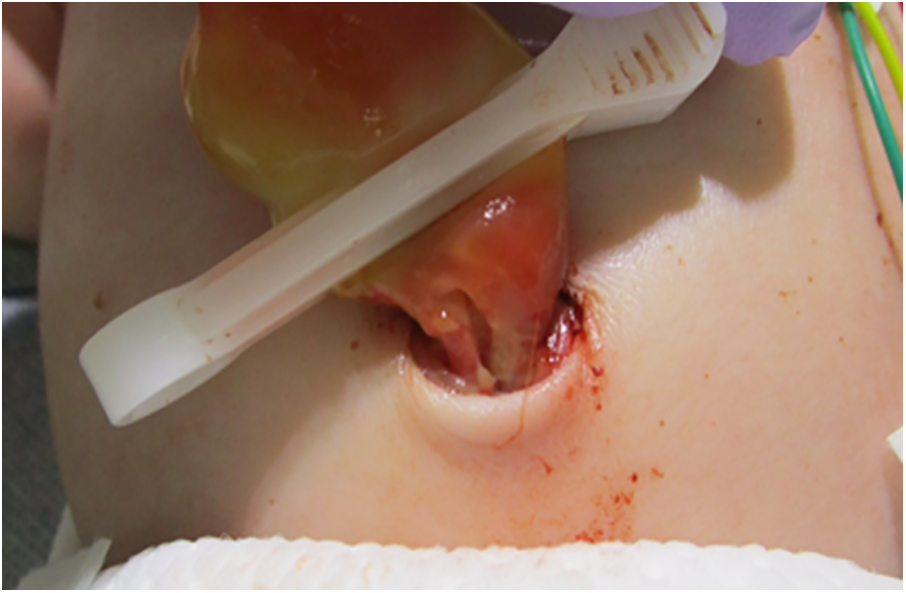
Site of fissure. The umbilical cord was partially tear close to the newborn and was bleeding arterially.

**Figure 2:**
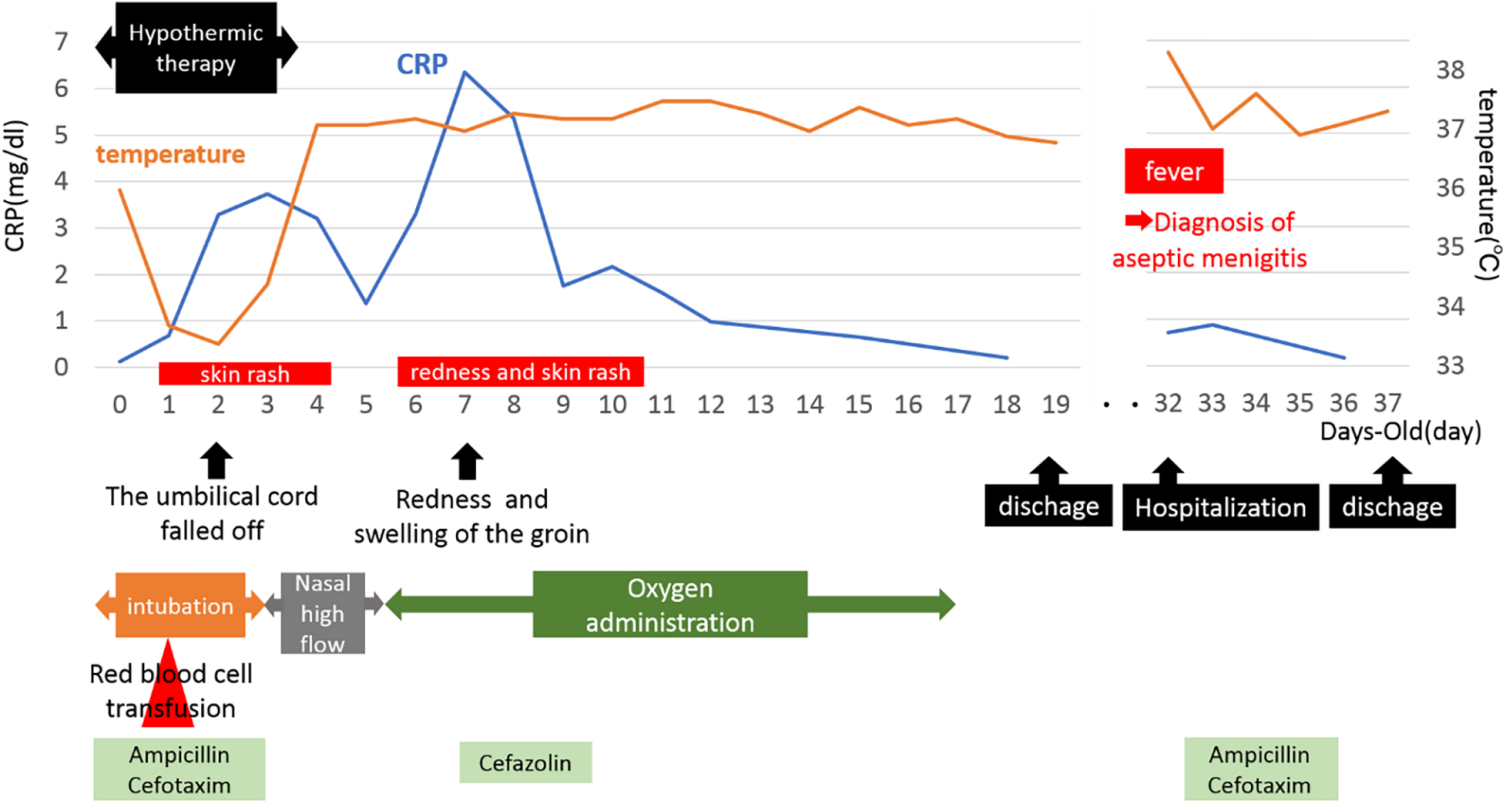
Patient course.

During delivery by cesarean section, a partial tear of the umbilical cord and arterial hemorrhage were observed at the site where the umbilical cord attached to the child (Figure 1). Manual compression was applied to stop the bleeding at the tear site while resuscitation efforts, including artificial respiration, were simultaneously performed. The baby was diagnosed with hypoxic-ischemic encephalopathy and underwent intensive care treatments, such as hypothermia therapy, in the NICU. Postnatal blood tests revealed anemia with a Hb level of 11.5 g/dl and a Ht of 33.9%, prompting a red blood cell transfusion (10 ml/kg). The umbilical cord appeared edematous, yellowish overall, very brittle, and detached on postnatal day 1. Head MRI scans performed on postnatal days 4 and 12, along with clinical findings after completion of hypothermia therapy, showed no abnormal neurological findings. During hospitalization, the baby had a generalized rash and recurrently elevated CRP levels of unknown cause, and antibiotics were used, although blood cultures remained negative. In addition, sudden swelling, and redness of the groin were noted on day 7, for which antibiotics treatment was administered. On postnatal day 31, after being discharged from the NICU, the baby developed aseptic meningitis and was re-hospitalized for treatment. The clinical course described above was consistent with CINCA syndrome/NOMID (Figure 2).

**Figure 3:**
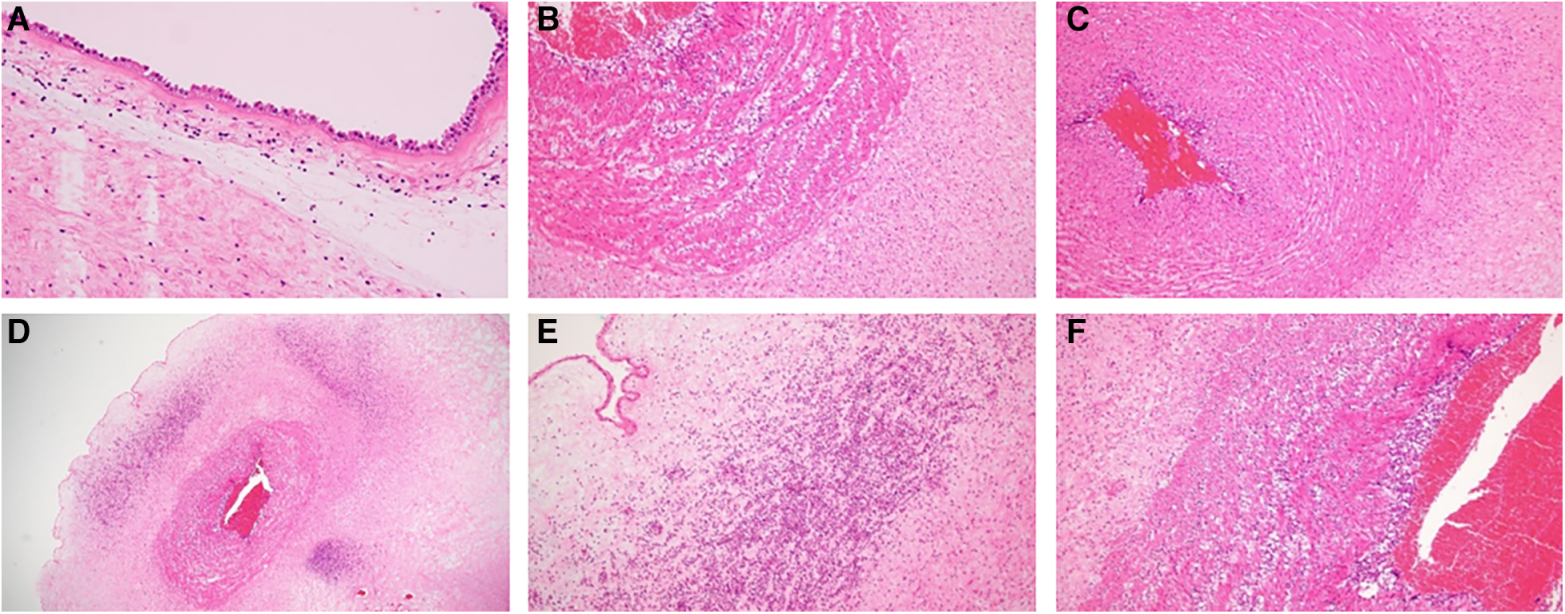
(**A**) Pathological photograph of the placenta. The placenta showed mild to moderate neutrophilic infiltration in the omentum, with a very small amount extending into the amnion. (**B**) Umbilical vein on the placental side. (**C**) Umbilical artery on the placental side. (**D**) Umbilical cord on the fetal side. Inflammatory cell infiltrate was stronger on the umbilical cord on the fetal side than on the placental side, and abscess formation was observed in Wharton's jelly. (**E,F**) Strong magnification of (**D**). Original magnification: (**B,C,F**) (×100), (**A,E**) (×200), and (**D**) (×20).

Pathological examination of the umbilical cord and placenta revealed stage III chorioamnionitis (Blanc classification) and stage III funisitis (Nakayama classification). The placenta showed mild to moderate neutrophilic infiltration in the omentum, with a very small amount extending into the amnion (Figure 3A). The umbilical cord was more significantly inflamed than the placenta, with inflammation extending into the veins, arteries, and Wharton's jelly. Inflammatory cells of the umbilical cord were more common in the umbilical artery than in the umbilical vein on the placental side (Figures 3B,C). Further, the infiltration of inflammatory cells in the umbilical cord was stronger on the fetal side than on the placental side. The vessel walls of the umbilical cord had coarse striations, cells were edematous, and abscess formation was observed in Wharton's jelly (Figures 3D–F). Inflammatory cells were predominantly MPO-positive cells, with a substantial number of CD68-positive cells also present. CD3-positive T cells and CD20-positive B cells were scarce.

Immunohistochemical analysis confirmed that these inflammatory cells were mainly neutrophils and macrophages, evidenced by their positivity for myeloperoxidase, with a subset also showing CD68 positivity.

The father, a teenager clinically diagnosed with CAPS, was a carrier of a disease-causing variant (*NLRP3* c.2068G>A p.Glu690Lys Hetero) (2, 5). Genetic testing of the baby revealed the same mutation as the father. This mutation is now known as the CINCA syndrome/NOMID (2). The baby was hospitalized once for episodes of fever, elevated CRP, and generalized rash that appeared to be symptoms of CINCA syndrome/NOMID., but resolved spontaneously. He will be started on canakinumab, a treatment for CAPS, after completion of live vaccines after the age of 1 year.

## Discussion

In this case, inflammation and rupture of the umbilical cord were the most likely causes of severe neonatal distress. There were no risk factors present for neonatal distress such as gestational hypertension, gestational diabetes mellitus, premature separation of the placenta, or intrauterine fetal growth restriction. Arterial hemorrhage at the site of umbilical cord attachment was observed during delivery by Caesarean section, and the rupture of the cord just before delivery was considered the cause of severe neonatal distress. Pathological analysis of the umbilical cord also showed strong inflammatory findings, particularly near the infant, with marked effects on Wharton's jelly and the vascular wall's muscular layer, indicating that the rupture was due to the cord's fragility caused by inflammation. The unusually rapid cord removal also suggests the vulnerability of the umbilical cord. In addition, umbilical cord rupture is generally reported as physical injury during vaginal delivery, umbilical cord omental adhesion, or injury due to umbilical cord ulceration associated with gastrointestinal atresia of the infant. In the present case, the baby was delivered by cesarean section and had no complications such as gastrointestinal obstruction.

The mother had no clinical signs of chorioamnionitis, IgM levels in the umbilical cord venous blood were not elevated, at 11 mg/dl, and no obvious intrauterine infection was present (6). With intrauterine infection, inflammation generally migrates from the placenta to the umbilical vein, followed by infiltration of the arteries and Wharton's jelly (7). The inflammation also spills over from the mother's side to the child's side. In this case, however, inflammation of the umbilical cord was more severe on the fetal side and the arterial side, suggesting that the cord inflammation may be due to inflammatory spillover from the fetus. Umbilical cord rupture is commonly attributed to physical injury during vaginal delivery, omental adhesion, or gastrointestinal obstruction in the infant (8–10). However, in this case, the baby was delivered via Caesarean section and had no complications such as gastrointestinal obstruction.

In general, hypothermia produces a suppression of inflammatory cytokine production and response and decreases various immune responses. Although multiple catheters are inserted during hypothermia and the patient is prone to infection, infection does not necessarily result in elevated white blood cell counts or CRP levels. In this case, CRP was elevated and more prolonged than in non-CAPS patients.

Neonatal symptoms of CINCA syndrome/NOMID include generalized redness and aseptic meningitis, yet reports of perinatal complications are scarce (11–13). Yokoi et al. reported a case of umbilical corditis, confined to the umbilical artery, in a preterm infant with CINCA syndrome/NOMID (14). This suggests that fetal inflammation caused tissue damage *in utero* and became the first manifestation of CINCA syndrome/NOMID in the neonate. The case presented here has a similar course and represents the first instance of umbilical cord rupture and severe neonatal distress in a full-term neonate, despite previous documentation of umbilical cord inflammation leading to preterm delivery. There have been no previous reports comparing the degree of inflammation on the infant's side with that on the placental side.

Given that fetal growth restriction and preterm delivery have been reported in CINCA syndrome/NOMID, it is plausible that inflammation beginning in the fetal period could persist and impact fetal development.

A limitation of this report is the lack of findings that robustly demonstrate an association between CAPS and umbilical cord inflammation. Intrauterine infection has also been reported to be associated with elevated inflammatory cytokines such as IL1β (15), making it difficult to distinguish between the two. It has also been suggested that the chronic CAM generally seen in late preterm and full-term infants may be due to non-infectious, immunologic causes (16). Arenas-Hernandez et al. suggested that activated T cells cause abnormal inflammation at the fetal-maternal interface without neutrophil infiltration (17). In this case, while lymphocyte infiltration is the main feature of amnion chorioamnionitis in normal-term infants (18), neutrophil, monocyte and macrophage infiltration was observed. Further, the strong inflammation on the infant's side of the umbilical cord was also unlikely to be the result of a normal intrauterine infection.

## Conclusion

In conclusion, this case involved cord rupture and severe neonatal distress in a patient diagnosed with CAPS, most likely stemming from corditis. It should be noted that births in individuals with a genetic predisposition to CAPS may be associated with complications related to placental and umbilical cord inflammation.

## Data Availability

The original contributions presented in the study are included in the article, further inquiries can be directed to the corresponding author.
